# A centralized global automation group in a decentralized organization

**DOI:** 10.1155/S1463924600000341

**Published:** 2000

**Authors:** James Ormand, Jimmy Bruner, Larry Birkemo, Judy Hinderliter-Smith, Jeffrey Veitch

**Affiliations:** 1 Glaxo Wellcome Research Triangle Park NC USA; 2 Glaxo Wellcome Hertfordshire Ware UK

## Abstract

In the latter part of the 1990s, many companies have worked to foster a ‘matrix’ style culture through several changes in organizational structure. This type of culture facilitates communication and development of new technology across organizational and global boundaries. At Glaxo Wellcome, this matrix culture is reflected in an automation strategy that relies on both centralized and decentralized resources. The Group Development Operations Information Systems Robotics Team is a centralized resource providing development, support, integration, and training in laboratory automation across businesses in the Development organization. The matrix culture still presents challenges with respect to communication and managing the development of technology. A current challenge for our team is to go beyond our recognized role as a technology resource and actually to influence automation strategies across the global Development organization. We shall provide an overview of our role as a centralized resource, our team strategy, examples of current and past successes and failures, and future directions.


Journal of Automated Methods & Management in Chemistry, Vol. 22, No. 6 (November–December 2000) pp. 195–198

### A centralized global automation group in a decentralized organization

**James Ormand[1], Jimmy Bruner[1], Larry Birkemo[1], Judy Hinderliter-Smith[1] and Jeffrey Veitch[2]**

*[1]Glaxo Wellcome, Research Triangle Park, NC, USA; [2]Glaxo Wellcome, Ware, Hertfordshire, UK*

*In the latter part of the 1990s, many companies have worked to foster a 'matrix' style culture through several changes in organizational structure. This type of culture facilitates communication and development of new technology across organizational and global boundaries. At Glaxo Wellcome, this matrix culture is re ected in an automation strategy that relies on both centralized and decentralized resources. The Group Development Operations Information Systems Robotics Team is a centralized resource providing development, support, integration, and training in laboratory automation across businesses in the Development organization. The matrix culture still presents challenges with respect to communication and managing the development of technology. A current challenge for our team is to go beyond our recognized role as a technology resource and actually to in uence automation strategies across the global Development organization. We shall provide an overview of our role as a centralized resource, our team strategy, examples of current and past successes and failures, and future directions.*

### Introduction

After the merger of Burroughs Wellcome and Glaxo, Inc., in 1995, a centralized robotics team was formed in the Development Information Services organization. This team was formed to support all departments in Development with close ties to both the Research and Manufacturing organizations. Prior to formation, automation resources were completely decentralized in the previous companies. Today at Glaxo Wellcome, we rely on both centralized and decentralized resources to develop and integrate automation.

The decentralized resources consist of automation experts located in each department. These experts focus on their particular business needs and help to coordinate automation efforts within their own organization. While experts are needed in each department, they are often not able to focus on global automation issues because of other responsibilities that they may have within their departments. The centralized automation team consists of four members; three in the US and one in the UK, and a manager in the US. Location within the Development IS organization positions the team as a core service for all departments within Development (figure 1).

The centralized team performs the same activities as the departmental automation employees with an emphasis on encapsulating common processes and transferring technology across departments. Team members were chosen based on their passion for automation, desired skill set (depth in different areas), and proven abilities in teamwork. One current challenge for this team is a perception that we still are not close enough to fully understand business needs. Another challenge is that we currently have no formal mechanism for interaction with the departments.

### Strategy

In the Development organization at Glaxo Wellcome, we rely on a strategy that is based on centralized and decentralized resources that 'mutually influence' each other. We accomplish this by the following.

- Developing partnerships with departmental leaders in automation. These leaders are familiar with combining the science with automation and will often become the business focus person in coordinating efforts of applying technology.
- Developing departmental leaders in automation by training/mentoring those that have an interest in automation. We have achieved this through 'borrowing' employees from departments for specific projects or for a predetermined amount of time. As these employees gain experience they also gain influence in leading automation efforts in their departments.
- Relying on management support, which is vital in ensuring success through communication and education of automation efforts at higher levels in the company. Department managers need to know about the resources that are available to them. Additionally, management support is needed to secure resources when needed.

### *Business principles*

- Task targeted automation: this is the concept of automating a single, well-defined task or a small group of tasks instead of automating an entire process. This has also been referred to as modular automation or the 'workstation approach' to automation. Applying this principle reduces development time, increases flexibility, lowers development/support costs, and facilitates duplication.
- Identify common activities: no automation project begins without thoroughly researching previous experience in the area of interest. This allows us to find the highest common denominator to start from. It also facilitates duplication if we can work at the highest level possible.
- Fully exploit vendor software/hardware: we choose this route whenever possible because it allows us to start with proven technology. We choose to extend the functionality of vendor supplied hardware/

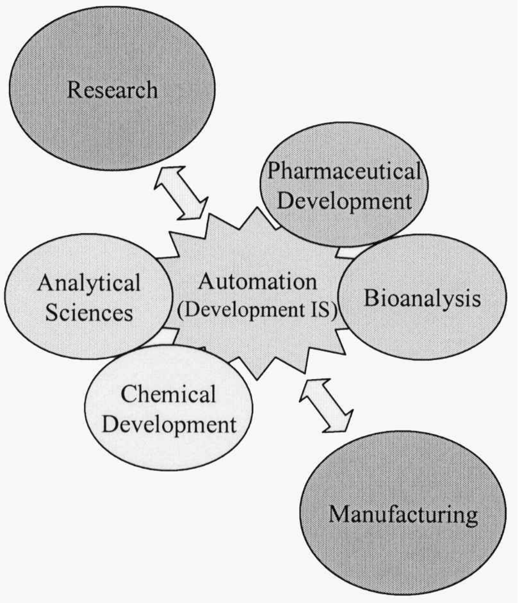

*Figure 1. Organizational view of the Centralized Automation Team.*

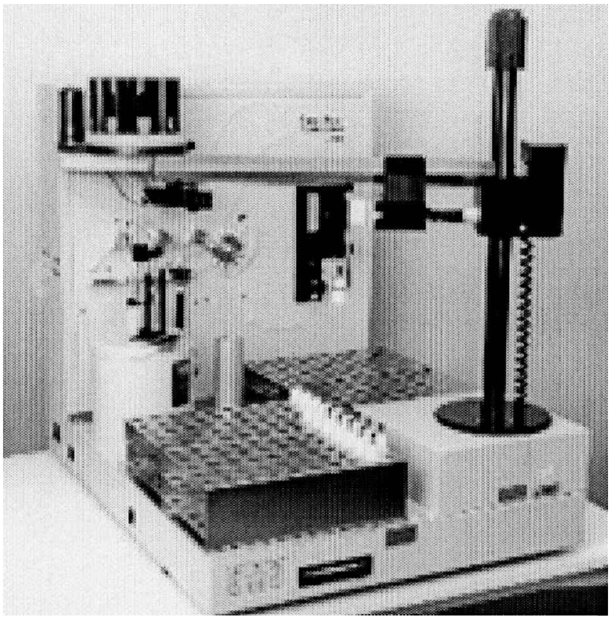

*Figure 2. Generic weighing application.*

software versus starting from scratch. This principle will ultimately assist vendors in product development.

- Prototype the proposed solution: this quickly exposes unexpected issues and allows for proof of concept.

### Examples

*Automation successes*

For our group, successful implementation of automation is defined as follows:

- documentation is complete;
- use of the application/system;
- replication of the application/system;
- positive feedback from users.

The following are examples of successful automation projects that have been completed/updated in the past year.

Platform:

BenchMate™ (Zymark Corp., Hopkinton, MA) (figure 2).

Highlights:

- reuse of unused equipment;
- simple, single process;
- replicated 7 times globally.

Platform:

Tecan Genesis (Tecan US, RTP, NC) (figure 3).

Highlights:

- common process for GxP labs;
- replicated 7 times globally.

Assay developed to use MDCK cells as an alternative to Caco-2 cells.

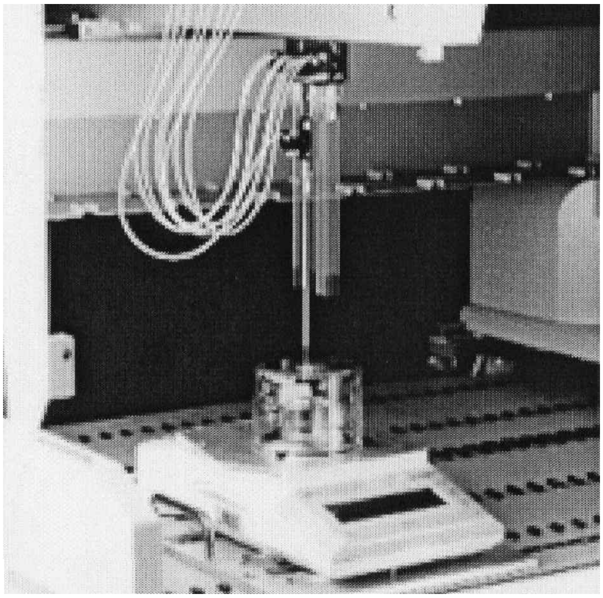

*Figure 3. Gravimetric verification of liquid handler performance.*

Platform:
Tecan Genesis Workstation (figure 4).

Highlights:

- new, patented technology: cells-on-sheets device [1];
- improved cell maintenance capabilities;
- designed with automation in mind;
- replicated 4 times globally.

Platform:

Gilson® 215 Liquid Handler (Gilson Inc., Middleton, WI) (figure 5).

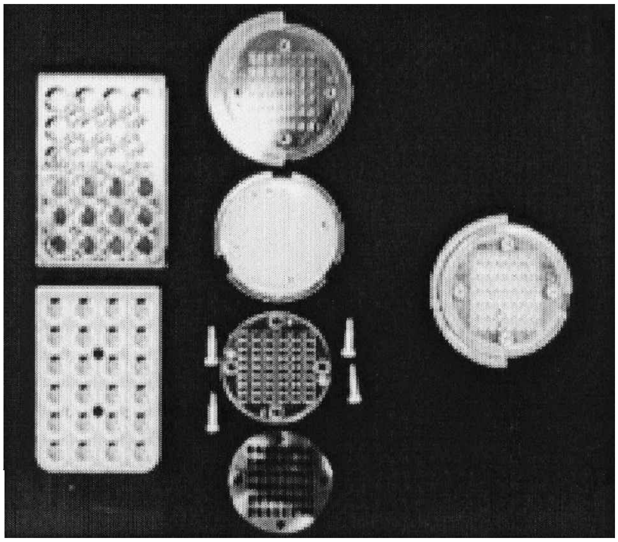

*Figure 4. Automation of intestinal absorption screen.*

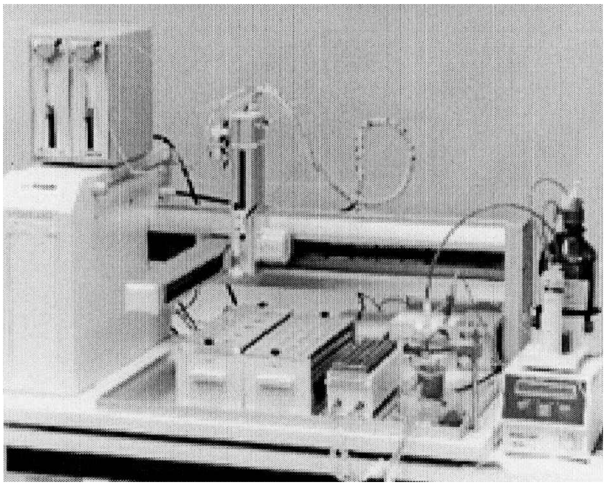

*Figure 5. Automation of pH solubility/stability screen.*

Highlights:

- cross-functional development;
- time savings of 1 h versus 2 days for manual process;
- data from system are in good agreement with historical results;
- will be replicated in 2000.

Other successful automation projects for our team this year are as follows.

- Joblist: Microsoft® Excel macro to extend the functionality of Tecan Logic software. This macro is currently used on 25 systems.
- Generic paging application for detection of instrument error and end events. This application is currently used on 10 systems.
- Tecan LUO: ewxtends functionality of PCS (Productivity, Communications and Scheduling Software, Zymark Corp., Hopkinton, MA) on Zymate® systems to run Tecan Logic and Gemini

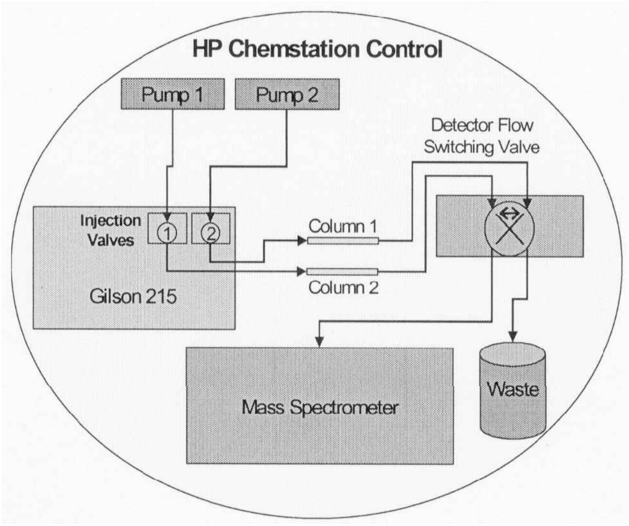

*Figure 6. High throughput LC/MS system.*

methods on Genesis Workstations. This LUO is currently used on 7 systems.
- Custom training: Negotiated a custom training class with Tecan for 19 Glaxo Wellcome employees.

*Current implementations*

We are also in the process of implementing several projects as follows.

Platform:
HP ChemStation (Hewlett-Packard GmbH, Waldbronn, Germany) (figure 6) and Gilson® 215 Liquid Handler (Gilson, Inc., Middleton, WI).

Highlights:

- will potentially double analysis throughput;
- cost savings of about $300 000 per installation;
- coordination of Chemstation macros with Gilson® modules.

Platform:
custom system developed by Spectrum Systems, LLC (Greenville, SC) (figure 7).

Highlights:

- partnered with outside vendor for development;
- uses technology from the manufacturing environment.

Other automation projects currently under implementation by our team this year are:

- addition of automated flow control of nitrogen on a Zymark 96-well evaporator;
- gravimetric tracking on liquid handlers;
- reaction optimization system.

### Stumbling blocks

In an effort to continually improve our ability to provide effective automation solutions, it is important for us to

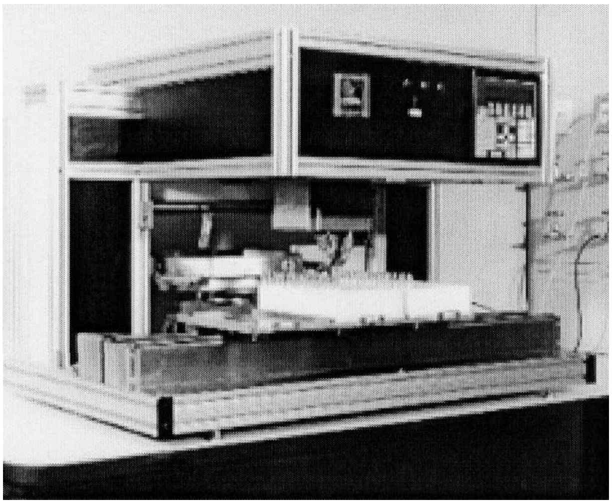

*Figure 7. Capping/uncapping system for samples from clinical sites.*

consider projects that have not been successful. Over the past several years, we have had four projects that were not completed. We have improved project delivery by focusing on the following.

- Quantify need: we now use tools such as cost/benefits analysis and user surveys to determine the value of a project.
- Fully understand process details: we strive even harder to learn as much as we can about the current process before starting an automation project.
- Avoid being 'too' creative: we focus on offering flexibility only where it is needed. This is referred to as 'non-restrictive standardization'.

### Future plans

- Enhance global communication: our location in the Development organization at Glaxo Wellcome puts us in a position to improve global communication in automation. Several potential vehicles for this include a development of a corporate 'knowledge network', newsletter, and our involvement in the Southeast Chapter of the Laboratory Robotics Interest Group (LRIG).
- Work with department leaders to refine processes: this will provide the groundwork to make automation feasible.
- Guide strategy in global processes: we are working to become involved in several global initiatives that are in need of a defined automation strategy.

### Acknowledgements

The authors wish to thank the following Glaxo Wellcome employees and automation companies for their contributions to all of the projects listed: David Igo, Phil Waters, Sonia Shamdasani, Chen Pingyun ('PY'), David Emiabata-Smith, Anita Simkins, Jeff Sailsad, Evelyn Barnes, Avis Bridgers, Glenn Smith, Bob Biddlecombe, Joe Long, Lloyd Frick, Cole Harris, Martin Owen, Affymax Research Institute, Spectrum Systems, LLC, Tecan, US, and Zymark.

### Trademarks

BenchMate™, PCS™, and Productivity, Communications and Scheduling Software™ are trademarks of Zymark Corporation (Hopkinton, MA).

Gilson® is a registered trademark of Gilson Inc. (Middleton, WI).

Microsoft® is a registered trademark of Microsoft Corporation (Redmond, WA).

### Reference

1. International patent number WO99/21958. Affymax Research Institute, 3410 Central Expressway, Santa Clara, CA, 95051, USA.

